# Bilateral geochemical asymmetry in the Karoo large igneous province

**DOI:** 10.1038/s41598-018-23661-3

**Published:** 2018-03-27

**Authors:** Arto V. Luttinen

**Affiliations:** Finnish Museum of Natural History, University of Helsinki, P.O. Box 44 FIN-00014 Helsinki, Finland

## Abstract

In the Karoo large igneous province, the geochemical assessment of mantle source variability and structure is hampered by probable crustal contamination overprinting of compositionally diverse flood basalts. Mantle source characteristics have been defined only for exceptional, primitive rock types. Here I use a compiled dataset for over 800 samples to demonstrate that the abundance of Nb relative to Zr, Ti, and Y provides a useful geochemical tracer of mantle sources for variably contaminated rock types of the Karoo province. Variations in the relative abundance of Nb reveal emplacement of distinctive, Nb-undepleted and Nb-depleted magmas in the North Karoo and South Karoo sub-provinces, respectively, and clarify correlation between flood basalts and previously proposed mantle source components. Judging from plate tectonic reconstructions and the compositions of plausible mantle source components, the geochemical bilateral asymmetry in Karoo may reflect tapping of contrasting plume and upper mantle reservoirs in the two sub-provinces.

## Introduction

Large igneous provinces (LIPs) are expressions of very large-scale mantle melting events that frequently relate to continental breakup, but are not explained by the plate-tectonic concept of magmatic activity. Melting of voluminous mantle plume heads is the most favoured generic model for LIP formation^1,2^, but, in many cases, the identity of the principal magma source remains elusive due to poor understanding of the geochemical structure of the mantle source region. This uncertainty presents a key problem in the study of terrestrial magmatism and mantle dynamics and composition.

Continental flood basalt (CFB) provinces are particularly problematic LIPs. While some continental LIPs can be geochemically associated with plume sources based on abundant ocean island basalt (OIB) -like rock types (e.g. Ethiopian Traps)^3^, many CFB provinces are characterised by isotopic and incompatible element ratios typical of continental crust. Whether or not this geochemical ‘crustal signature’ results entirely from crustal contamination of plume-sourced magmas^4^ or represents a primary feature derived from non-plume sources in subcontinental lithospheric mantle (SCLM)^5^ or subduction-modified convective upper mantle^6^, or from exotic plume components^7^ has been a long-standing controversy.

Thermodynamically constrained, quantitative models of magmatic differentiation^8^ suggest that even minor contamination with crustal or SCLM wall-rock melts can have a controlling influence on the incompatible element and isotopic ratios of CFBs^9^. This means that the crustal signature of many CFBs is very likely to stem at least partially from contamination overprinting. Due to many uncertainties related to geochemical modelling, it is very difficult to distinguish source-derived features of contaminated basalts and only exceptional, compositionally primitive rock types facilitate detailed isotopic and incompatible element characterisation of the mantle components involved. Resolving the geochemical structure of the mantle sources in continental LIPs thus calls for correlation of voluminous CFBs with such mantle components. This exercise is critically dependent on identification of geochemical tracers that hold information on the primary magmas and mantle sources even in the case of strongly contaminated CFBs.

**Figure 1:**
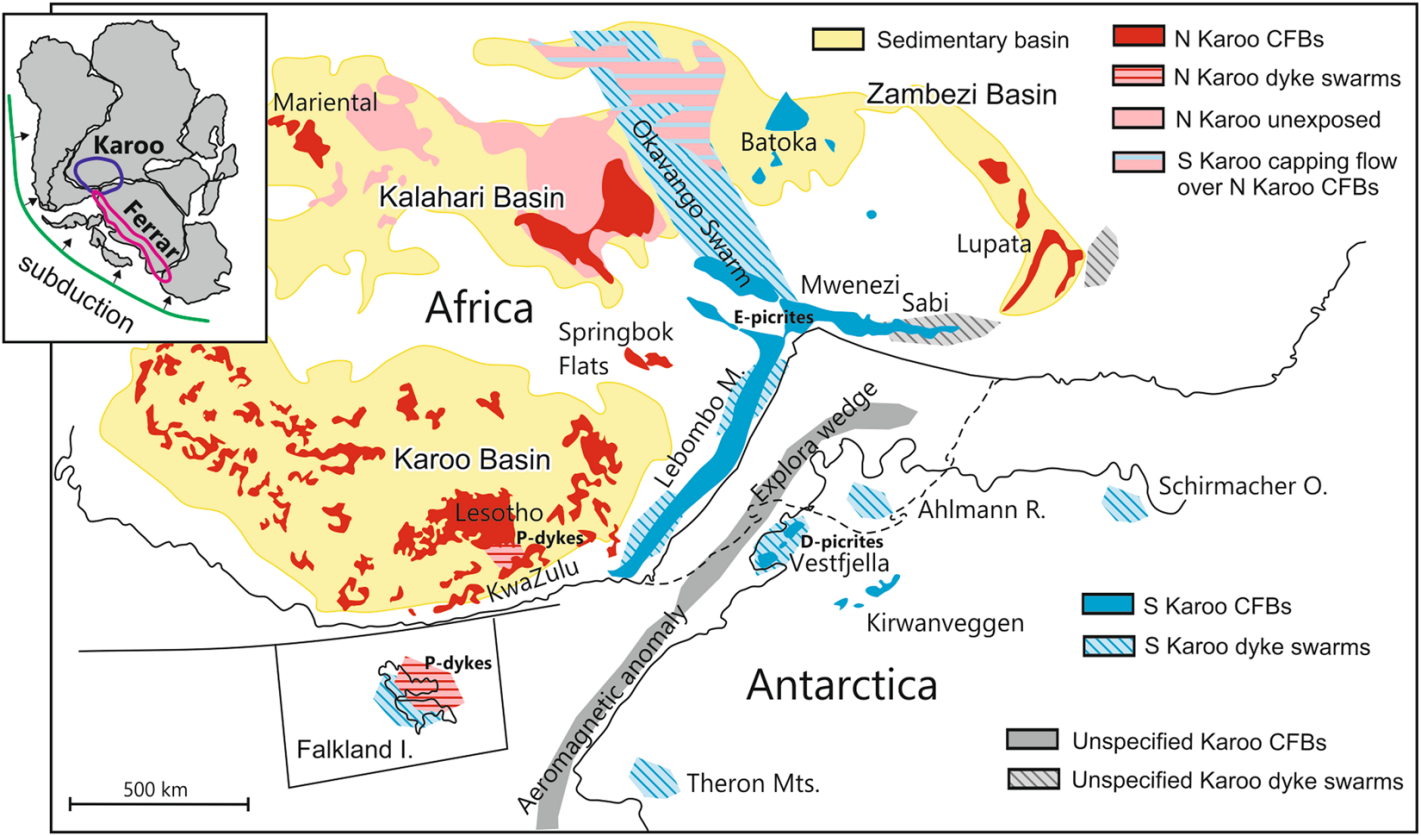
Distribution of Karoo CFBs and related intrusive rocks in Mid-Jurassic Gondwana reconstruction. The North Karoo and South Karoo sub-provinces are indicated by red and blue colours, respectively. The seaward-dipping lava successions (Lebombo Monocline, Sabi, Vestfjella), radiating dyke swarms (Okavango, Sabi, Lebombo Monocline, Vestfjella, Ahlmannryggen) and submarine seismic reflectors (Explora Wedge)^66^ and aeromagnetic anomalies^67^ define the Karoo triple rift^12,34^. Picrite suites that show geochemical affinities to depleted mantle (D-picrites)^23^ and enriched lithospheric mantle (E-picrites)^18,20,25,28^, and basaltic dykes with (non-chondritic) primitive mantle^50^ -like compositions (P-dykes)^44,48^ may represent significant parental magma types. Karoo Basin CFBs include the Lesotho lava succession and a large sill complex^37^. The inset shows distribution of the Karoo CFBs relative to the coeval Ferrar CFBs and an active subduction zone along Gondwana margin^58^. The occurrences of Karoo igneous rocks are after refs^18,66^. The map was created using CorelDraw (https://www.coreldraw.com/). The outlines of continents were drawn using vector maps by FreeVectorMaps.com (http://freevectormaps.com) as models and the igneous formations were drawn after Luttinen *et al*. (ref.^64^) and Jourdan *et al*. (ref.^18^) with publisher’s permission.

The Mid-Jurassic Karoo CFB province represents a prime example of the mantle source problem in geochemically complex LIPs. The Karoo magmatism in southern Africa and East Antarctica at ca. 180–183 Ma^10,11^ was contemporaneous with the formation of the Ferrar CFB province during the initial stages of Gondwana breakup (Fig. 1). A plume origin for Karoo has been previously argued based on, for example, evidence of regional uplift and extension patterns^12,13^ and indications of high mantle temperatures^14^, short duration and large scale of magmatism^11^, and reconstructed palaeopositions of modern hotspots^15^. In contrast, the diverse geochemical crustal signatures of the Karoo CFBs have been frequently linked to generation of the magmas from different kinds of SCLM sources^16–18^. Bearing in mind the possibility of strong crustal contamination overprinting^9,19^, the variable CFB compositions may provide little information on the mantle sources, however. The geochemical characteristics of primary magmas and mantle sources in the Karoo LIP have been constrained in studies of picritic rock types that preserve primary compositional features. These are also compositionally notably variable and point to melting of SCLM^20,21^ and different recycled lithospheric^22–25^ and subduction fluid-influenced components^23^ in the convective upper mantle.

The genetic relationships of the diverse flood basalts and picrites and the general geochemical structure of the mantle sources in Karoo have been addressed by grouping of various magma types into strongly enriched (high-Ti) and mildly enriched (low-Ti) categories based on TiO_2_ and Zr/Y (Supplementary Figs. S1 and S2). The high-Ti and low-Ti categories have been conventionally regarded to reflect two fundamentally different magmatic assemblages derived from distinctive mantle sources^18,26,27^. It has become clear, however, that the Karoo picrite data do not corroborate a simple high-Ti vs. low-Ti mantle source provinciality. The picrite suites reveal that (1) high-Ti and low-Ti compositions can be produced from common primary sources simply because of variable degree of melting^9,23^, or by variable hybridisation with secondary lithospheric sources during magma ascent^28^, and (2) at least high-Ti magmas originated from several geochemically different primary sources in SCLM and the underlying convective mantle^21,23,25,29^. Few of the Karoo picrites have been correlated with specific compositional types of CFBs and it is presently disputable whether the identified mantle components represent all significant magma sources and which one of them, if any, represents the predominant mantle source for Karoo LIP. Overall, it is uncertain to what extent the diversity of CFB compositions records mantle source heterogeneity, whether lithospheric or convective mantle sources were predominant, and whether a mantle plume was involved.

Here I examine the geochemical structure of the Karoo LIP using a compiled set (n = 819) of previously published data and new chemical analyses (n = 98) on Karoo CFBs (Methods; Supplementary Data). I suggest the abundance of Nb relative to other high field strength elements (Zr, Ti, Y) to represent a useful petrogenetic tracer for the Karoo LIP. A division of Karoo into relatively Nb-depleted southern and Nb-undepleted northern sub-provinces (in 180 Ma Gondwana reconstruction; Fig. 1) provides a new framework for geochemical, geophysical, and geochronological research on the Karoo LIP and is suggestive of a large-scale bilateral geochemical structure in the mantle source regions that were influenced by coeval subduction and mantle plume activity.

## Results

### Geochemical asymmetry revealed by Nb-Ti-Zr-Y chemistry

**Figure 2:**
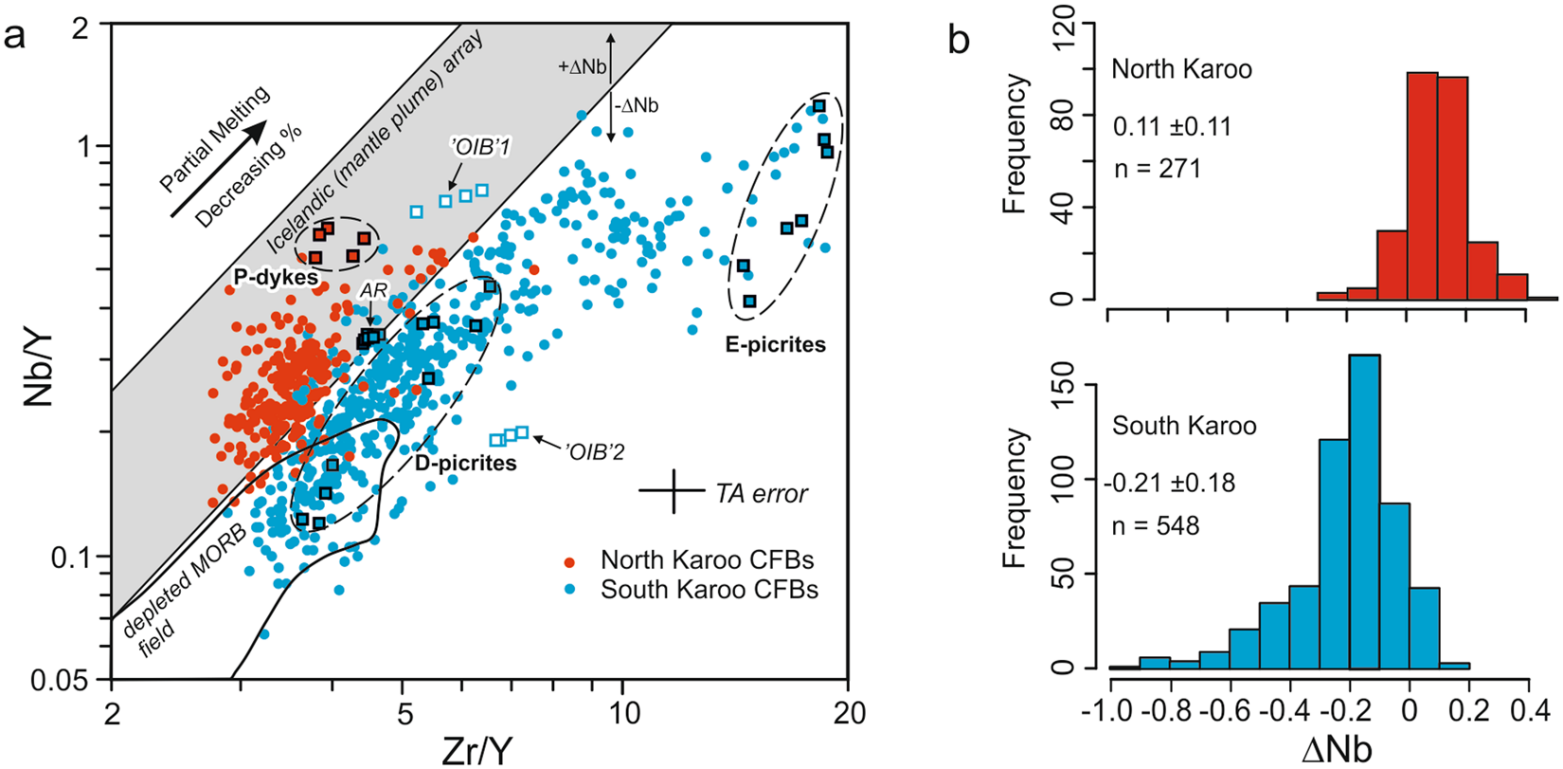
Relative abundances of Nb, Zr, and Y in Karoo CFBs. (**a**) Variation of Zr/Y and Nb/Y in Karoo CFBs relative to depleted MORB and Icelandic plume-derived volcanic rocks. Plausible parental magma types with MORB-like (D-picrites^23^ and Ahlmannryggen (AR)^29^ dykes for South Karoo CFBs; P-dykes^44,48^ for North Karoo CFBs) and SCLM-like (E-picrites^18,20,25,28^ for South Karoo) characteristics are indicated and compositions of OIB-like South Karoo dykes^23,29^ are shown for comparison. The deviation of Nb/Y from the lower limit of the Icelandic array is quantified using a ΔNb parameter^31^ and TA error indicates estimated total analytical error (Methods). (**b**) Frequency distribution, average and standard deviation of ΔNb values. MORB data from http://georoc.mpch-mainz.gwdg.de/georoc/ and Icelandic array from ref.^31^.

The abundance of Nb relative to other incompatible elements has been recognized as a key character of mantle reservoirs^30^. Comparison of Nb/Y and Zr/Y values is an effective tool for addressing the issue in continental settings as other frequently used parameters (e.g. Nb/U and Nb/La) are more strongly affected by contamination. In the Nb/Y vs. Zr/Y approach, basalts are often compared to the compositional field of Icelandic volcanites^31^. The excess or deficiency in Nb relative to the lower limit of the Icelandic array can be quantified using a ΔNb parameter^31^ (Fig. 2; see Methods). Generalizing, normal (depleted) mid-ocean ridge basalts (MORBs) have relatively Nb-depleted compositions (negative ΔNb), whereas ocean island basalts (OIBs and MORBs generated at enriched ridge segments exhibit Nb-undepleted compositions (positive ΔNb) (Fig. 2) and have been associated with mantle plume-related magmatism^31–33^. From the viewpoint of CFB mantle sources, it is crucial to note that ΔNb is an isotope-like parameter, i.e. it is only weakly affected by variable degrees of fractional crystallisation or mantle melting^33^ and that the continental crust is Nb-depleted (http://georoc.mpch-mainz.gwdg.de/georoc/). Accordingly, positive ΔNb values are very likely to indicate Nb-undepleted mantle sources, whereas negative ΔNb values can result from Nb-depleted mantle sources or extensive crustal contamination.

In Karoo LIP, a bimodal distribution of ΔNb values for geographically distinctive sub-provinces reveals an outstanding bilateral asymmetry:

Radiating dyke swarms and seaward-dipping lava successions along the African and Antarctic margins outline a triple rift structure in the middle of the pre-breakup Karoo province^12,34^ (Fig. 1). The CFBs associated with the Karoo triple rift and those of the East Antarctic plateau escarpment are designated here as South Karoo (Fig. 1). In the Zr/Y vs. Nb/Y space, the isotopically heterogeneous (initial ε_Nd_ at 180 Ma mainly +4 to −10) and chemically highly variable low-Ti and high-Ti basalts and picrites of South Karoo (e.g. refs^17,18,29,35^) (Supplementary Fig. S2) define a broad array with relatively low Nb/Y at given Zr/Y and mainly plot below the plume array with negative ΔNb (Fig. 2). Their relative Nb-depletion is also indicated by low Nb/Ti and Nb/Zr values at given Zr/Y (Supplementary Fig. S3; see Methods). In comparison, North Karoo is composed of the widespread low-Ti CFB lavas, sill complexes, and minor dyke swarms found across and in the proximity of extensive Permian-Jurassic sedimentary basins (i.e. Karoo, Kalahari, Zambezi; Fig. 1; Supplementary Fig. S2). They are in many respects much more uniform (ε_Nd_ mainly −1 to −4) than the South Karoo CFBs, although some incompatible element ratios are equally variable^18,36,37^. Importantly, the North Karoo CFBs are characterized by higher Nb/Y (positive ΔNb) as well as Nb/Ti and Nb/Zr at given Zr/Y than the South Karoo compositions (Fig. 2; Supplementary Fig. S3). Bearing in mind the great diversity of incompatible and isotopic ratios in the Karoo CFBs, the bimodality of the ΔNb values (Fig. 2b) with remarkably restricted geographical and geochemical overlap (Fig. 1 and Supplementary Note) manifests fundamentally different origin for the North Karoo and South Karoo CFBs.

### Influence of crustal contamination on ΔNb

**Figure 3:**
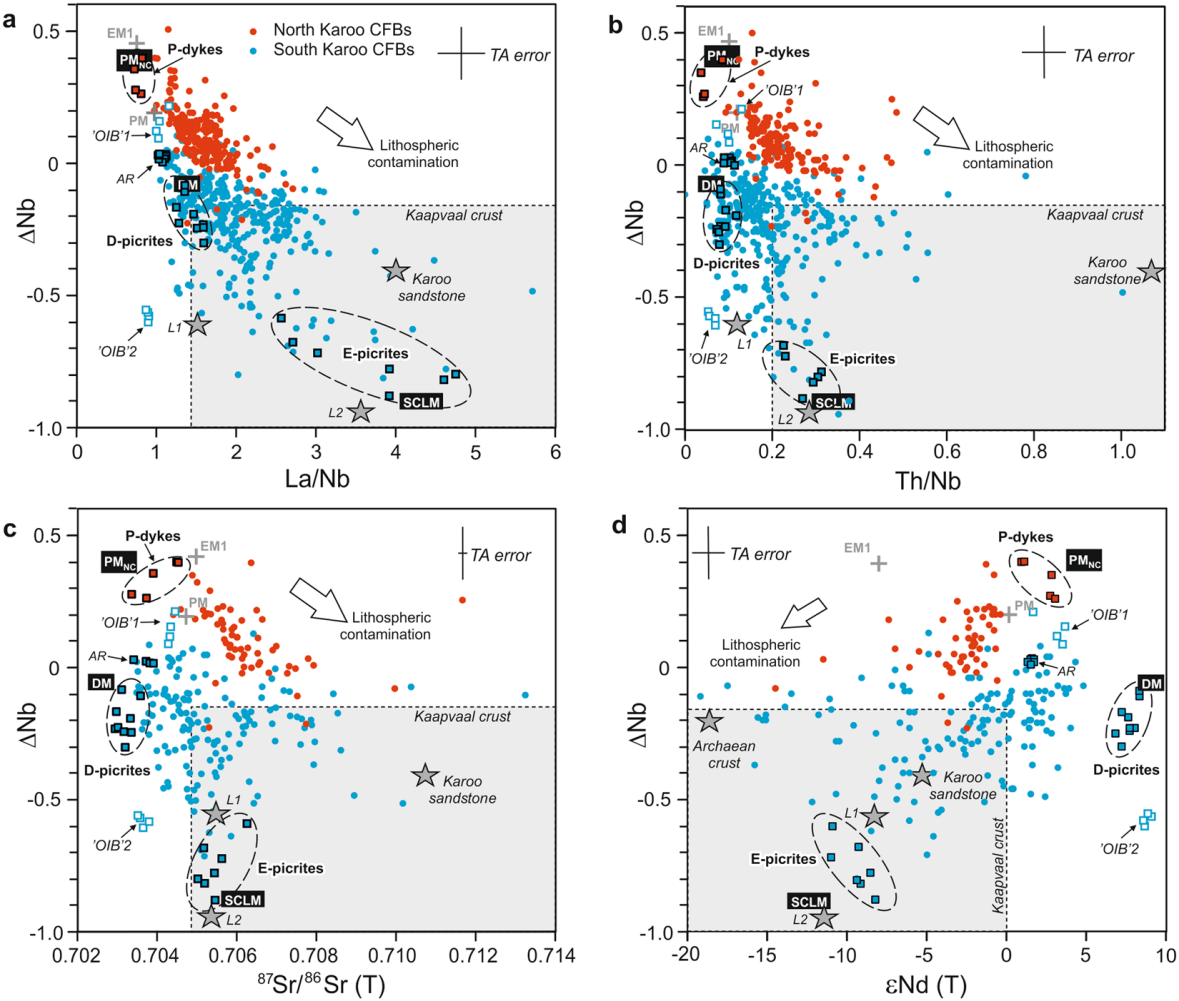
Correlation between ΔNb and contamination-sensitive incompatible element and isotopic ratios. ΔNb values versus (**a**) La/Nb, (**b**) Th/Nb, (**c**) initial ^87^Sr/^86^Sr and (**d**) initial ε_Nd_ in Karoo CFBs. Plausible parental magma types with MORB-like (D-picrites^23^ and Ahlmannryggen (AR)^29^ dykes for South Karoo CFBs; P-dykes^44,48^ for North Karoo CFBs) and SCLM-like (E-picrites^18,20,25,28^ for South Karoo) characteristics are indicated. Compositions of possibly related mantle source components (depleted mantle DM^68^; estimated South Karoo SCLM; chondritic primitive mantle PM^69^; non-chondritic primitive mantle PM_NC_^49,50^; enriched mantle EM1 based on Walvis Ridge from http://georoc.mpch-mainz.gwdg.de/georoc/) are illustrated. Compositions of OIB-like South Karoo dykes^23,29^ are shown for comparison. The influence of lithospheric contamination and compositions of possible lithospheric contaminants are schematically indicated [silicic rocks of Kaapvaal crust from http://georoc.mpch-mainz.gwdg.de/georoc/; Karoo sandstone^37^; Archaean crust^18^; SCLM-sourced melts L1 (Vestfjella lamproite, South Karoo)^64^ and L2 (Leucite Hills lamproite, Wyoming)^65^. TA error is estimated total analytical error (Methods). The isotopic compositions are calculated at 180 Ma, apart from L1 (160 Ma), and DM, PM, PM_NC_, and EM1 reservoirs and L2 (present-day values).

Extensive crustal contamination can impose negative ΔNb values on originally Nb-undepleted basalts, whereas the opposite is very unlikely due to preponderance of Nb-depleted compositions in crustal rock types^31,33^. The degree of crustal contamination can be evaluated using Th/Nb, La/Nb, and initial ^87^Sr/^86^Sr and ε_Nd_ values as contamination indexes (Fig. 3). Generalising, crustal contaminants tend to have variably high ^87^Sr/^86^Sr and negative ε_Nd_ and they are very likely to have high Th/Nb and La/Nb (Fig. 3; http://georoc.mpch-mainz.gwdg.de/georoc/). The great majority of Karoo CFBs show geochemical indications of crustal contamination. In the case of North Karoo, correlations in Fig. 3 indicate that crustal contamination tends to drive CFB compositions towards lower ΔNb values and negative ΔNb are mainly associated with the most strongly contaminated basalts. The prevalence of positive ΔNb values indicates Nb-undepleted parental magmas and mantle sources in North Karoo.

In South Karoo, the ΔNb values are mainly negative (Fig. 2) and overlap with typical values of continental crust (Fig. 3). However, the contamination indexes Th/Nb, La/Nb, ^87^Sr/^86^Sr, and ε_Nd_ are suggestive of predominantly similar or even lower degrees of crustal contamination in South Karoo relative to North Karoo (Fig. 3; Supplementary Fig. S4). Very low ε_Nd_ are more common in the South Karoo CFBs and relate to exceptional contaminants (Archaean crust or SCLM), rather than exceptionally high degree of contamination^9,16,19,28^. These observations indicate that the crustal contaminants were systematically notably more Nb-depleted across South Karoo, or that the South Karoo parental magmas and mantle sources were Nb-depleted. Several lines of evidence favour mantle origin for the negative ΔNb values in South Karoo. First, the age, composition, and thickness of large-scale crustal units (e.g. craton vs. off-craton) in southern Africa and Antarctica^38–40^ do not correlate with the CFB provinciality (compare Fig. 1 and Supplementary Fig. S2), which renders geochemically distinct contaminants in the two sub-provinces unlikely. Second, the Nb-depleted South Karoo CFBs include a high abundance of rock types that are nearly uncontaminated based on very low Th/Nb, La/Nb, and ^87^Sr/^86^Sr and high ε_Nd_ (Fig. 3; Supplementary Fig. S4), which proves involvement of Nb-depleted mantle sources in South Karoo. Third, the ΔNb values show poor correlation with crustal contamination indexes and there are no indications of high-ΔNb parental magmas in the South Karoo CFB data. Fourth, the nearly uncontaminated and variably contaminated Nb-depleted basalts are stratigraphically intercalated and record a continuous compositional range in the South Karoo data^17,19,29,35^. Fifth, crustal contamination models lend support to generation of South Karoo CFBs from Nb-depleted sources^9,19^.

Importantly, the compositions of feasible parental magma types of Karoo CFBs similarly fall into Nb-depleted and Nb-undepleted types (Figs. 2 and 3), which helps to link variably contaminated CFBs to previously identified mantle components.

### South Karoo from Nb-depleted sources

Previous studies of Karoo mantle sources have been effectively limited to South Karoo, where primitive, picritic rock types indicate several types of mantle components. Two high-Ti picrite suites from Antarctica lack geochemical crustal signature and show broad geochemical similarities to OIB (‘OIB’1 and ‘OIB’2 in the figures)^23,29^. They are compositionally very different from each other and from the South Karoo CFBs (Figs. 2 and 3) and have been considered to represent melting of minor components of recycled oceanic crust in convective mantle^19,23,24^. The OIB-affinity picrites have been regarded to be implausible parental magma types for voluminous CFB magmatism^19,23,24^, and are excluded from this study (Supplementary Note).

On the other hand, two Nb-depleted, but otherwise geochemically contrasting picrite suites may represent significant parental magma types of South Karoo CFBs. One of them is composed of the voluminous, incompatible element-enriched picrite lavas of the Letaba formation at the Karoo triple junction (e.g. ref.^28^) (Fig. 1). These geochemically variable (e.g. ε_Nd_ −4 to −11), predominantly high-Ti type picrites have been have been widely regarded to be parental to isotopically similar, but more evolved high-Ti Karoo CFBs^18,35^ and to represent melting of heterogeneous SCLM^18,20,21^ or recycled lithospheric material in convective mantle^22,25^. However, the wide compositional range of the Letaba picrites records strong evidence for mixing of magmas from two contrasting mantle reservoirs^18,20,28,41,42^. The most enriched Letaba picrites (E-picrites) represent a magma type which exhibits a potassic character and incompatible element and isotopic (Sr, Nd, Pb, Os) ratios approaching those of SCLM-derived lamproite magmas^28,41^ (Fig. 3). Many studies have considered that the E-picrites and the Letaba formation in general were generated by mixing of relatively depleted parental magmas with lamproite-like low-degree partial melts from SCLM^18,28,29^. In the mixing models of the Letaba picrites, the depleted endmember has been tentatively identified as a MORB-like magma from convective mantle^18,28,29^ and the estimated amount of the lamproite-like enriched endmember from SCLM ranges from few up to 40 wt.% (in E-picrites)^28^.

The other Nb-depleted picrite suite that may represent an important parental magma type of South Karoo CFBs is located at Vestfjella, in the Antarctic part of South Karoo (Fig. 1). This suite of low-Ti to high-Ti basalt and picrite dykes (D-picrites; depleted ferropicrites and low-Nb dykes of ref.^23^) is isotopically (Sr, Nd, Pb, Os, He) indistinguishable from the depleted upper mantle (DM)^23,43^ (Fig. 3) and is a possible example of the MORB-like endmember predicted in the mixing model of the Letaba picrites^28^. Quantitative geochemical models for the Antarctic part of South Karoo support a significant role for DM magma sources by showing that the D-picrites may represent a parental magma type which evolved to diverse low-Ti and high-Ti CFB types in Vestfjella by fractional crystallisation, contamination with crust, and mixing with SCLM melts^9,19^.

Overall, the large geochemical variations of the Nb-depleted South Karoo CFB types can be explained quite well by interaction between three principal components: (1) DM sampled by the D-picrites, (2) SCLM sampled by the E-picrites, and (3) continental crust (Fig. 3). In South Karoo, the ΔNb values are characteristically negative because all of these three isotopically different mixing components are Nb-depleted (Fig. 3). The occurrence of subordinate South Karoo rock types with mildly positive ΔNb, such as isotopically mildly depleted MORB-like dykes at Ahlmannryggen (Figs. 1–3), is suggestive of compositional variation in relatively Nb-depleted convective upper mantle (Supplementary Note). Geochemical models indicate, however, that DM is the best candidate for a common primary source of both low-Ti and high-Ti magma types in the South Karoo sub-province^9,19,28^. Enriched SCLM^18,20,21,28^ (or recycled lithospheric material)^22,25^ is likely to be a significant source for the most enriched magma types (i.e. E-picrites and resembling high-Ti basalts) (Fig. 3).

### North Karoo from Nb-undepleted sources

In North Karoo, picrite suites have not been reported and the mantle sources of this geographically more extensive sub-province have not been well constrained. In the past, most geochemical studies have ascribed the different compositional types of North Karoo CFBs^36^ to melting of heterogeneous SCLM^16,18,26^, but the subtle trace element and isotopic variations across North Karoo could have been caused by crustal contamination overprinting of broadly uniform parental magmas^37,44^. Judging from the least-contaminated CFB compositions, the North Karoo CFBs require Nb-undepleted (ΔNb of ca. +0.3) parental magmas with isotopic and incompatible element ratios resembling those of primitive mantle (PM) (Fig. 3). Such parental magmas are quite different from the D-picrites and E-picrites of South Karoo.

Theoretically, the parental magmas of North Karoo CFBs could have been generated by extensive melting of geochemically PM-like SCLM sources. Within the area of the northern sub-province, compositional data from kimberlite-hosted mantle xenoliths and highly potassic rock types reveal isotopically (e.g. ε_Nd_ +4 to −12)^45,46^ and chemically (ΔNb −0.18 to 0.87)^47^ profoundly heterogeneous SCLM which may well contain geochemically plausible sources for North Karoo CFBs. If the parental magmas of North Karoo had been derived entirely or partially from heterogeneous southern African SCLM, they would be expected to exhibit compositional diversity on provincial scale. It is therefore important to notice that the coherent geochemical trends for North Karoo CFBs suggest parental magmas that were notably uniform with regard to Nd and Sr isotopic compositions and ΔNb values (Fig. 3). Generation of uniform parental magmas from SCLM would have required homogenization of compositionally diverse magma batches in remarkably well-mixed, stable, and nearly identical plumbing systems across the province, or very long distance transportation of magmas from a single centre of extensive SCLM melting and magma processing. Bearing in mind the huge scale of magmatism in North Karoo (Fig. 1), I consider that such efficient homogenisation of magmas is unlikely. Given that generation of voluminous, dry low-Ti CFB magmas principally from re-fertilised, hydrous SCLM has been considered improbable^4^, I maintain that a large and relatively homogeneous convective mantle reservoir is a geochemically and thermodynamically most likely environment for production of parental magmas for voluminous, uniform North Karoo CFBs.

**Figure 4:**
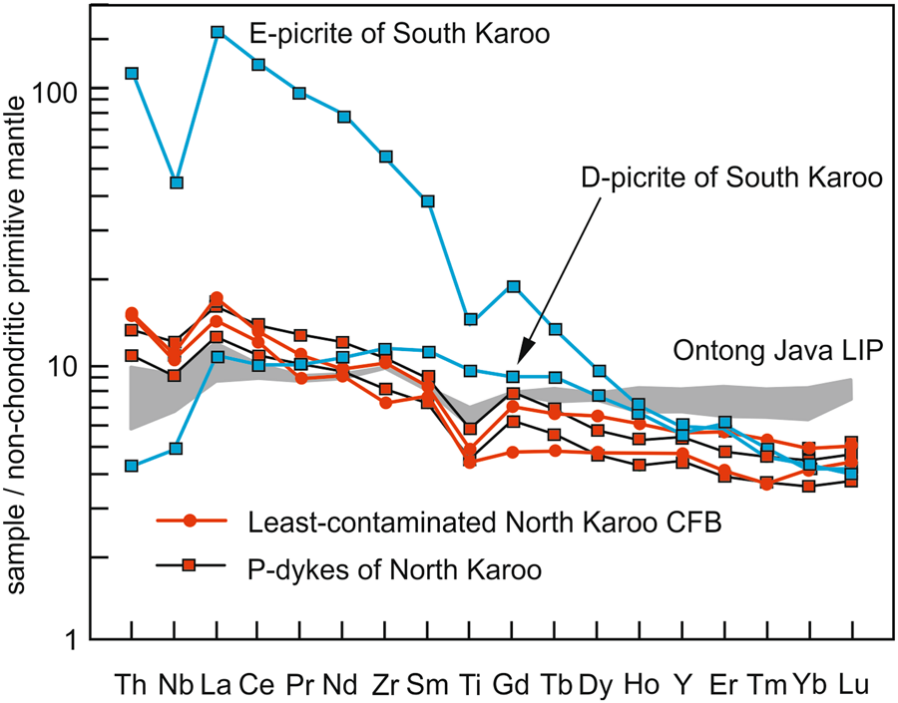
Incompatible trace element compositions of parental North Karoo magmas normalised to non-chondritic primitive mantle^50^. Compositions of the least-contaminated CFBs (samples KO4-C-55 and OXB-54) and MORB-like dykes (P-dyke samples SA.19.1 and SA.20.1) from North Karoo show overall similarity and resemble basalts of the Ontong Java LIP (representative Kroenke and high-Nb type samples)^70^. Different incompatible element contents in Karoo and Ontong Java samples can be ascribed to different melting conditions. The Ontong Java LIP has been associated with melting of non-chondritic primitive mantle plume material (akin to PM_NC_ in Fig. 3) rising from deep mantle^49^. Compositions of parental South Karoo magmas (D-picrite sample P27-AVL and E-picrite sample N356) are shown for comparison.

Interestingly, recently reported c. 180 Ma dykes in KwaZulu-Natal, SE Africa^44^, and geochemically quite similar, undated dykes in the Falkland Islands (Mount Alice Type in ref.^48^) (Fig. 1) may provide insights into North Karoo mantle source. These minor suites include dykes that lack geochemical indications of crustal contamination (e.g. high La/Nb and Th/Nb) and resemble MORB^44,48^. In contrast with South Karoo, however, the MORB-affinity rocks of North Karoo (P-dykes) have positive ΔNb (ca. +0.3) and show mildly depleted initial isotopic compositions (ε_Nd_ +1 to +4; ^87^Sr/^86^Sr 0.7035–0.7045) (Figs. 2 and 3) and their primitive mantle-normalised incompatible element patterns are markedly different from those of D-picrites and E-picrites (Fig. 4). Importantly, the isotopic and incompatible element compositions of the P-dykes resemble those of least-contaminated North Karoo CFB samples (Figs. 3 and 4), which suggests generation from a geochemically similar mantle source. Relatively high Nb/Y and Zr/Y (Fig. 2) and low heavy rare earth element contents (Fig. 4) in the P-dykes point to lower degree of mantle melting in the case of the dyke magmas.

In the discourse on LIPs and global mantle reservoirs, PM-like incompatible element ratios and isotopically mildly depleted compositions similar to those of the P-dykes are typical of many oceanic plateau basalts (e.g. Ontong Java LIP) and have been associated with primitive mantle plume sources (so-called non-chondritic primitive mantle; Figs. 3 and 4)^49,50^. If the P-dykes of North Karoo were derived from the same source as the North Karoo CFBs, an argument for a plume source can be presented based on the great size, high emplacement rates^11^, and uniform Nb-undepleted compositions of the North Karoo CFBs, as well as the overall geochemical similarities to (non-chondritic) primitive mantle plume sources (Figs. 3 and 4).

## Discussion

The division of the Karoo LIP into Nb-depleted and Nb-undepleted sub-provinces provides a new framework for geological, geochronological, and geophysical research.

First, classification based on relative Nb abundances helps to understand why the conventional high-Ti vs. low-Ti division seems to work in some parts of the Karoo LIP and leads to ambiguities in other parts. For example, in southern Botswana the intercalated low-Ti and high-Ti CFBs belong to North Karoo and South Karoo provinces, respectively, and geochemical data are indicative of fundamentally different origins for these categories^18^. In contrast, the intercalated low-Ti and high-Ti CFBs in Lebombo monocline and the Antarctic rifted margin show overlapping characteristics^19,35^, because they may have been derived from the same overall South Karoo mantle source.

Second, evaluation of the high-precision age data reveals that the U/Pb zircon and baddeleyite dates are almost exclusively limited to North Karoo (ref.^11^ and references therein) and the ^40^Ar/^39^Ar plagioclase ages also show a similar provincial bias (ref.^10^ and references therein). Overall, reliable age data indicate rapid emplacement of CFBs across the North Karoo basins at ca. 182–183 Ma^10,11^, which is also compatible with palaeomagnetic observations^51^. In contrast, U/Pb and ^40^Ar/^39^Ar ages in South Karoo are fewer and effectively limited to crosscutting intrusions^10,19^ and silicic volcanic rocks at high stratigraphic levels^10,52^ in the Karoo rift: The available data prove contemporaneous magmatic activity in both sub-provinces, but they do not provide firm age constraints for South Karoo. In fact, the palaeomagnetic observations^51^ and ^40^Ar/^39^Ar ages^10,19^ show implications of earlier onset of magmatic activity in the southern sub-province, although the age data are sparse. Bearing in mind the contrasting geochemical characteristics of South Karoo and North Karoo (Figs. 2–4), it is possible that also the onset, frequency, rate, and duration of magma emplacement in the two sub-provinces were notably different.

Third, while geophysical research has frequently pointed to involvement of mantle plume in Karoo LIP (e.g. refs^2,15^), geochemical studies have favoured non-plume sources and influence of subduction-modified upper mantle in particular (e.g. refs^23,25,43^). This controversy may stem from the fact that previous geochemical research of Karoo mantle sources has been focused on South Karoo picrites^20–25,28,29^ (Fig. 1) and that the North Karoo CFBs may have been derived from a fundamentally different mantle domain.

**Figure 5:**
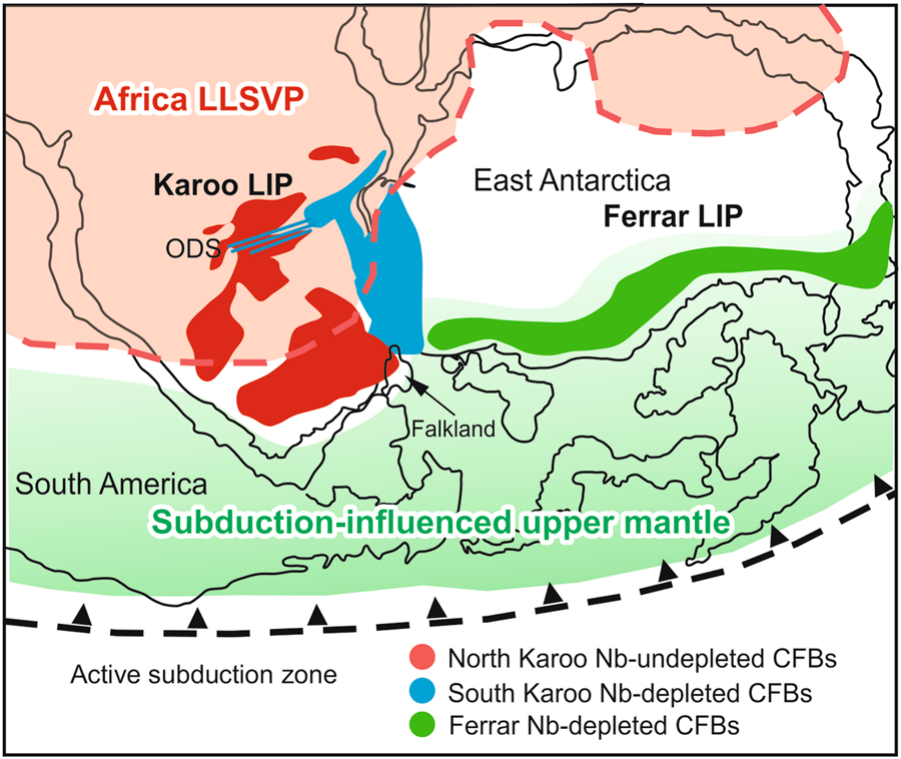
Distribution of Nb-undepleted and Nb-depleted Karoo CFBs relative to the sub-African large low-shear velocity province (LLSVP)^15^, the southern Gondwanan subduction zone^58^, and the Ferrar LIP. The surface projection of LLSVP boundary at 2800 km depth relative to reconstructed Gondwana is shown in red^15^ and possible extent of subduction-modified depleted upper mantle is schematically indicated with light green. The bilateral geochemical asymmetry in Karoo LIP is consistent with tapping of plume sources within and subduction-influenced upper mantle sources outside the realm of the African LLSVP. Ferrar LIP has been frequently associated with subduction-influenced upper mantle sources (e.g. ref.^55^) and is characterised by negative ΔNb values (Supplementary Fig. S5). The map was created using CorelDraw (https://www.coreldraw.com/). The overall Gondwana reconstruction and the outline of African LLSVP is based on Torsvik *et al*. (ref.^15^) and the outlines of continents were drawn using vector maps by FreeVectorMaps.com (http://freevectormaps.com) as models.

It is presently uncertain whether the distribution of the Nb-depleted and Nb-undepleted CFBs corresponds to a similar bilateral geochemical structure in the mantle, or results from magma emplacement patterns. The occurrence of Nb-depleted CFBs along the Okavango dyke swarm probably exemplifies long-distance lateral transportation of magmas^34^ under controlling influence of (possibly pre-Karoo) lithospheric structures^53^ (Supplementary Note; Supplementary Fig. S2). Nonetheless, it is interesting to notice that the observed bilateral CFB provinciality could be linked to the large-scale mantle convection pattern beneath Jurassic Gondwana (Fig. 5). A hypothesis of magma production under the influence of active zones of subduction and plume upwelling provides a framework for combining previous contradictory ideas into a unifying conceptual model for Karoo LIP.

Several previous studies have brought up the possibility that the predominant mantle source of Karoo and some other CFB LIPs was subduction-influenced upper mantle^6,27,54,55^. While the general geochemical crustal signature of Karoo CFBs is compatible with subduction-modified mantle sources^6,27,55^, it is not specific to subduction and can result entirely from crustal contamination, as demonstrated by recent geochemical models^9,37^. Picritic rock types of South Karoo provide geochemical evidence for subduction-modified mantle sources, however: The D-picrites of South Karoo lack indications of crustal contamination and are isotopically indistinguishable from modern MORB along the Southwest Indian Ridge^23,43^. Nevertheless, they record variable enrichment of fluid-mobile elements relative to other incompatible elements (e.g. Ba/Th up to 300% PM values)^23^ and contain hydrous primary mineral inclusions in olivine phenocrysts^56^. While the exact causes of these characteristics are presently unclear, addition of subduction fluids into the mantle source is a viable mechanism. Furthermore, geochemical traces of recycled oceanic crust in other types of South Karoo picrites^21,23–25^ and strong selective enrichments of fluid-mobile elements in South Karoo flood basalts at the Antarctic rifted margin^57^ may also be associated with a variably subduction-modified reservoir in convective upper mantle. Given the relative proximity of South Karoo to active subduction along the paleo-Pacific Gondwana margin^58^, it is possible that subduction-affected material was transported to the regions of CFB magma generation^6^ (Fig. 5). The South Karoo CFBs could thus have tapped the same overall subduction-modified region of depleted upper mantle as that widely advocated for the coeval, but more strongly subduction-influenced Ferrar CFBs^6,55^ which are also typified by negative ΔNb values (Fig. 5; Supplementary Fig. S5).

In comparison, the geochemical compositions of the least-contaminated CFBs and the P-dykes of North Karoo do not indicate subduction-modified sources and are compatible with an isotopically mildly depleted mantle plume source, instead. Many recent studies have concluded that the Karoo LIP was formed at a time when the region was positioned over the southern limit of the sub-African deep mantle seismic anomaly, one of Earth’s two detected large low shear-velocity provinces (LLSVPs) (e.g. ref.^15^). Several research groups have the opinion that the margins of LLSVPs correspond to plume starting zones (e.g. ref.^59^). Judging from plate tectonic reconstructions, a large part of the North Karoo sub-province formed over the LLSVP (Fig. 5). It seems plausible that melting of an ascending mantle plume from the LLSVP margin could have facilitated rapid generation of voluminous North Karoo parental magmas which evolved to compositionally different types of CFBs largely due to crustal contamination. It is also worth noticing that LLSVPs have been regarded to be likely reservoirs of non-chondritic primitive mantle material^49^ so that the geochemical implications of non-chondritic mantle source in North Karoo (Figs. 3 and 4) comply with a plume model. Finally, involvement of a hot mantle plume source in North Karoo would help to understand signs of Jurassic regional uplift in southern Africa^12,13^ and could explain indications of unusually high temperatures for the upper mantle source of South Karoo in the Vestfjella region^60^ adjacent to the provincial boundary (Fig. 1).

The proposed scenario for geochemically complex magmatism in the Karoo LIP associates the observed CFB provinciality to interplay of active zones of subduction and plume upwelling. Broadly similar ideas of plume-subduction interaction have been previously presented (e.g. ref.^61^), but the conventional high-Ti vs. low-Ti classification of CFBs has indicated inconsistent (i.e. concentric or radial) geochemical provinciality (Supplementary Fig. S2). The discovery of the geochemical bilateral asymmetry using relative abundances of Nb, Zr, Ti, and Y provides new observational evidence of compositional zonation predicted by models of combined plume and plate-boundary driving forces and is consistent with preferential tapping of plume sources within and upper mantle sources outside the realm of the African LLSVP. Most importantly, the geochemical dichotomy in CFBs may help to reconcile apparently contradictory previous results and provides a new framework for geoscientific research on the Karoo LIP.

## Methods

### New geochemical data for Karoo CFBs

This study reports previously unpublished data on major and trace elements for 98 samples of Karoo CFBs. High-precision trace element data for some key localities (e.g. Lesotho and southern Lebombo; Fig. 1) have been previously lacking. In order to complement the trace element dataset, I selected a set of 51 samples from southern and central Lebombo (South Karoo; Fig. 1), originally reported by Duncan and co-workers (ref.^62^) and Sweeney and co-workers (ref.^35^), for reanalysis at the Peter Hooper Geoanalytical Laboratory, Washington State University, using XRF and ICP-MS methods. The results are given in Supplementary Table S1. Additionally, geochemical data on 47 lava samples from the Lesotho area (North Karoo; Fig. 1), similarly analysed using XRF and ICP-MS at the Geoanalytical Laboratory and originally listed in the unpublished PhD thesis of Jakub Rehácek (ref.^63^), are included in Supplementary Table S2 with the permission of Dr Rehácek. For sample descriptions and information on general geochemical characteristics, the reader is referred to refs^35,62,63^. Importantly, comparison with a large unpublished dataset on Lesotho CFBs (600 samples; Goonie Marsh personal communication, 2007) suggests that the elemental geochemical data for 47 Lesotho samples (North Karoo) are representative of the >1 km thick basalt succession.

References for the analytical methods at the GeoAnalytical Laboratory are given in Supplementary Table S3 and http://cahnrs.wsu.edu/soe/facilities/geolab/technotes/. The results for international standards indicate good accuracy and precision (<3%) for major and trace elements in general (Supplementary Table S3). Analyses of 256 duplicate samples (two aliquots of the same sample) from a wide range of geological materials, and spanning three instrumental re-calibration cycles, yield relative percent difference values [RPD = (│Duplicate A − Duplicate B│)/(Duplicate A + Duplicate B)/2) × 100%] for the XRF method at the Geoanalytical Laboratory. The RPD values for incompatible element-poor samples provide a conservative estimate of practical precision for the XRF methodology, including sample preparation, and suggest precision of ca. 1% for Zr, ≤4% for Y, and ≤7% for Nb. In the case of the ICP-MS data, repeated measurements of an incompatible element-poor standard suggest precision of ≤2% for Zr, ≤2% for Y, and ≤5% for Nb. Comparison indicates good agreement between XRF and ICP-MS data and high correlation coefficients for Zr (r^2^ = 0.999), Y (r^2^ = 0.998), and Nb (r^2^ = 0.998) (Supplementary Tables S1 and S2).

### Compiled geochemical dataset for Karoo

This study is based on a compiled dataset of 819 geochemically analysed samples representing the Karoo LIP (Supplementary Data). The dataset includes published data and previously unpublished data for 98 samples reported in this study. Overall, the compiled geochemical dataset now covers the Karoo LIP rather well. Published geochemical data for the CFBs in the Kalahari and Zambezi basins and the Sabi Monocline are sparse and compositional data for the submarine CFBs inferred from seismic data along the rifted margins are not available, however (Fig. 1).

The samples in the compiled dataset can be divided into low-Ti (n = 266 North Karoo, n = 269 South Karoo), high-Ti (n = 155 South Karoo), and transitional-Ti (n = 4 North Karoo, n = 125 South Karoo) basalts, basaltic-andesites, and picrites, with high-Ti compositions identified based on TiO_2_ > 2 wt.%^18^ and Zr/Y > 6^26^ (Fig. 1; Supplementary Fig. S1). The compiled dataset includes published Sr (n = 223) and Nd (n = 217) isotopic data as well as unpublished Sr and Nd isotopic data (n = 16) from ref.^63^.

The geochemical Karoo data have been analysed at various laboratories over a long period. High-precision ICP-MS data on trace elements (including REE, Th, Nb and Y) are available for 69% (n = 562) of the samples and have been preferably used for the compiled dataset and the geochemical plots of this study. The ICP-MS data are mainly from the Geoanalytical Laboratory (n = 38 North Karoo and n = 254 South Karoo), Chemex Laboratories (n = 32 North Karoo and n = 113 South Karoo), University of Durham (n = 15 North Karoo and n = 71 South Karoo), and University of London (n = 113 North Karoo). Examination of geochemical data from these laboratories shows that the variations in ΔNb are large (mainly >0.6 units) compared to typical analytical precision (ca. ± 0.07 units based on 2σ errors at the Geoanalytical Laboratory and refs^18,29,37^) and confirms that North Karoo and South Karoo exhibit different ranges for ΔNb. Inter-laboratory bias cannot be determined using the available published data. Consequently, the error bars for Nb/Y (±0.03), Zr/Y (±0.3), Nb/Ti (±0.0001), Nb/Zr (±0.01), ΔNb (±0.1), La/Nb (±0.3), and Th/Nb (±0.05) in Figs. 2, 3, and Supplementary Fig. S3 are based on estimated total analytical error (bias of standard analyses + precision) of ≤1% for Ti, ≤5% for Zr, ≤8% for Y, ≤10% for Nb and La, and ≤20% for Th (see Supplementary Table S3 and refs^18,29,37^). These values are likely to represent a conservative error estimate for the majority of Karoo samples with the possible exception of very incompatible element-poor samples. The error estimates for initial ^87^Sr/^86^Sr (±0.0001) and ε_Nd_ (±1 unit) in Fig. 3 are based on refs^17-19,23,29,37^ and include ca. 20% uncertainty in the ^87^Rb/^86^Sr and ^147^Sm/^144^Nd values.

### Evaluation of mantle sources using relative abundances of Nb, Zr, Ti, and Y

The relative abundances of Nb, Zr, Ti, and Y in basalts depend mainly on source chemistry, degree of melting, and possible incorporation of lithospheric wall-rock material. The influence of fractional crystallisation on the relative abundances is generally small and is limited to the most evolved types which have been affected by removal of clinopyroxene or Ti-Fe oxides, or both. The ΔNb value is an empirically developed geochemical tool for quantifying the abundance of Nb relative to Zr and Y^31^. It is based on the observation that Nd isotopically depleted Icelandic, presumably plume-related volcanic rocks can be distinguished from depleted MORB from the northern Atlantic Ocean by their systematically higher Nb/Y at given Zr/Y^31^. The Icelandic compositions are taken to reflect coupled variations of Nb/Y and Zr/Y during variable melting processes of a broadly uniform mantle source, so that large variations in Nb/Y at the same Zr/Y are suggestive of geochemically different magma sources. In a logarithmic plot of Nb/Y and Zr/Y, the Icelandic data define a linear array. The enrichment or deficiency of Nb relative to the lower limit of the Icelandic (plume) array (Fig. 2) is quantified using ΔNb values (Equation 1)^31^.

1 $${\rm{\Delta }}\text{Nb}=1.74+\,\mathrm{log}({\rm{Nb}}/{\rm{Y}})-1.92\,\mathrm{log}({\rm{Zr}}/{\rm{Y}})$$

Examination of global data has revealed that OIB and other oceanic basalts related to hotspots generally have high-ΔNb (Nb-undepleted) compositions in contrast with the low-ΔNb (Nb-depleted) characteristics of normal MORB ref.^32^. Numerical modelling shows ΔNb values of basalts to be only weakly affected by variable degrees of mantle melting^31,33^. Prolonged fractional crystallisation of Ca-pyroxene will cause mild decrease in ΔNb values in evolved magmas^32^.

In this study, the relative Nb abundances have been examined using the ΔNb method^31^. The Nb abundances have been also evaluated by comparing the Karoo CFBs with Icelandic volcanic rocks in Nb/Ti vs. Zr/Y and Nb/Zr vs. Zr/Y diagrams (Supplementary Fig. S3). Variations of Nb/Ti and Nb/Zr at given Zr/Y have been quantified using ΔNb_Ti_ values (Equation 2) and ΔNb_Zr_ values (Equation 3) in similar fashion as ΔNb.

2 $$\,{{\rm{\Delta }}\text{Nb}}_{{\rm{Ti}}}=3.85+\,\mathrm{log}({\rm{Nb}}/{\rm{Ti}})-1.23\,\mathrm{log}({\rm{Zr}}/{\rm{Y}})$$

3 $${{\rm{\Delta }}\text{Nb}}_{{\rm{Zr}}}=1.55+\,\mathrm{log}({\rm{Nb}}/{\rm{Zr}})-0.73\,\mathrm{log}({\rm{Zr}}/{\rm{Y}})$$

The slope in equations 2 and 3 has been derived from log(Zr/Y) vs. log(Nb/Ti) and log(Zr/Y) vs. log(Nb/Zr) plots of Icelandic data using linear regression and the constant has been determined by visual fitting. Fractional crystallisation of Ti-bearing pyroxene and oxides tends to increase ΔNb_Ti_ values in evolved basalts. The analytical errors in the Nb, Zr, Ti, and Y data correspond to uncertainty of ca. ±0.07 in ΔNb_Ti_ and ca. ±0.06 in ΔNb_Zr_.

In continental setting, the ΔNb values of basalts can be significantly influenced by incorporation of crust or SCLM material. However, due to the Nb-depleted composition of continental crust, crustal contamination is not expected to increase ΔNb and is very unlikely to produce Nb-undepleted magmas from Nb-depleted parents^31^. Many studies have assumed that lamproites provide the best proxy for SCLM-sourced contaminants (e.g. refs^18,28,29^). Geochemically, they are characterised by very high contents of incompatible elements and negative ΔNb values^28,64,65^ so that SCLM-contamination is generally expected to generate low ΔNb values (Fig. 3). However, lamproite-like rock types in southern Africa frequently have high ΔNb values^47^ and incorporation of such SCLM-derived components to Karoo basalts would have led to high ΔNb. Bearing in mind that the lamproite-type rocks in southern Africa are younger than Karoo LIP^47^, there is a distinct possibility that North Karoo CFBs in fact generated a high-ΔNb SCLM component rather than vice versa. Notably variable ΔNb values in the uncontaminated and mildly contaminated rock types (Fig. 3) and coupling of the ΔNb values with the geographic distribution of the CFBs (Figs. 1 and 2) suggest ΔNb (and ΔNb_Zr_ and ΔNb_Ti_) are promising tools for mapping of large-scale mantle heterogeneity in the Karoo LIP. Due to relatively high uncertainties associated with inter-laboratory bias, stratigraphic relationships and detailed geochemical characteristics (magma types)^17,19,29,36,37,44^ are crucial in consideration of small differences in the ΔNb values (<0.2 units).

### Data availability

The dataset generated during this study and the compiled geochemical data that support the findings of this study are included in this published article (Supplementary Tables S1–S3; Supplementary Data).

## Electronic supplementary material

Supplementary Information

Dataset 1a

Dataset 1b

Dataset 1c

Dataset 2
