# Supplementary material for: Bilateral geochemical asymmetry in the Karoo large igneous province
Source: Sci Rep. 2018 Mar 27;8:5223. doi: 10.1038/s41598-018-23661-3 (PMC5869710; doi:10.1038/s41598-018-23661-3)
Supplement: Supplementary file 1 — Supplementary Information [file 41598_2018_23661_MOESM1_ESM.pdf]

Bilateral geochemical asymmetry in the Karoo large igneous province

by Arto V. Luttinen

SUPPLEMENTARY INFORMATION

Supplementary Note: Definition of North Karoo and South Karoo sub-provinces

In this study, the abundance of Nb relative to other incompatible elements, specifically Zr, Ti, and Y, has been used to present a new geochemical provincial scenario for the Karoo LIP. The division into South Karoo and North Karoo sub-provinces is primarily geographical:

Generalizing, South Karoo includes the East-Antarctic portion of the Karoo LIP (Vestfjella, Kirwanveggen, Ahlmannryggen, Schirmacher Oasis, Theron Mountains) as well as the lavas and intrusions of the Karoo triple rift zone (Lebombo Monocline, Mwenezi, Sabi, Okavango dyke swarm) (Fig. 1). North Karoo includes widespread lavas, sill complexes and minor dyke swarms associated with the Permian-Jurassic sedimentary basins of southern Africa (Karoo, Kalahari, and Zambezi in Fig. 1). Comparison between these two geographically defined sub-provinces reveals a bimodal distribution of DNb (Fig. 2) and DNb_{Ti} values, and differences in DNb_{Zr} values (Supplementary Fig. S3) with North Karoo typified by positive and South Karoo by negative values.

Overall, with regard to DNb values, the CFBs of North Karoo and South Karoo show remarkably limited compositional overlap. Examples of compositional and spatial overlap are discussed below.

Compositional overlap. The most significant case of compositional overlap is represented by North Karoo CFBs that have negative DNb values (Figs. 2–3 and Supplementary Fig. S3). However, these samples also exhibit high La/Nb, Th/Nb and initial $^{87}Sr/^{86}Sr$, and low initial eNd, and their Nb-depleted compositions thus most likely reflect strong crustal contamination (Fig. 3).

Some of the South Karoo CFBs exhibit positive DNb. It should be noted, however, that these South Karoo samples largely represent a volumetrically minor suite of dyke rocks from Ahlmannryggen (Group 2 of ref 1) and are overrepresented in the dataset. These dykes are relatively enriched in incompatible elements (transitional-Ti) (Supplementary Fig. S1) and could record a parental magma type of some South Karoo CFBs. I tentatively ascribe them to heterogeneity within the Nb-depleted

upper mantle source of South Karoo, possibly due to mixing of high-DNb and low-DNb mantle source components close to the provincial boundary (Fig. 1).

Spatial overlap. In contrast, the occurrences of low-DNb lavas in Springbok Flats [2], Batoka [3] (Victoria Falls) and adjacent to the Okavango dyke swarm in the Zambezi Basin (capping flow atop of thick North Karoo CFB suite [4]) represent spatial overlap: i.e. they exhibit strong geochemical affinity to South Karoo, but are spatially intercalated with North Karoo CFBs.

These high-Ti and transitional-Ti CFBs have been interpreted to manifest transportation of South Karoo magmas into the northern sub-province (Fig. 1 and Supplementary Fig. S2). The justification for this decision stems from the universal low-Ti-affinity of the North Karoo CFBs (Supplementary Fig. S1) and the fact that high-Ti and transitional-Ti CFBs are strongly associated with the Karoo triple rift, i.e. the southern sub-province (Supplementary Fig. S2). Bearing in mind the high-Ti (and low-DNb) character [3,4] and the WNW magma flow direction [5] of the Okavango dykes (Supplementary Fig. S2), it seems quite possible that the parental magmas of the Okavango dykes were generated within the triple junction area (Mwenezi). It is thus possible that the Okavango swarm represents a feeder system of the rare examples of low-DNb CFBs outside the Karoo triple rift pattern.

On the other hand, Karoo-related dykes on the Falkland Islands [6] have been designated to North Karoo and South Karoo based on their DNb values and are regarded to represent intercalation the sub-provinces adjacent to the triple rift (Fig. 1).

Excluded OIB-affinity dykes. Sparse, strongly incompatible element-enriched high-Ti dykes show broad geochemical similarities to OIB in South Karoo. A subgroup of high-Ti dykes in Ahlmannryggen (Fig. 1; Group3 of ref. 1) has low-DNb typical of South Karoo, but is geochemically quite different from any other Karoo rock type ('OIB'2 in Figs. 2-3). On the other hand, the enriched ferropicrites [7] and compositionally resembling basalt dykes (high-Nb category of ref 8) represent rare examples of high-DNb rocks in Vestfjella, South Karoo, and plot apart from Karoo CFBs in the figures ('OIB'1 in Figs. 1-2). Judging from their isotopic (Nd, Sr, Pb, Os) compositions and generally different geochemical compositions compared to Karoo CFBs, the OIB-like dykes probably represent subordinate enriched mantle components [7–9] and they are not included in the compiled data.

Excluded Ferrar-affinity samples. Finally, it should be noted that the basal part of the thick Lesotho lava succession, well within the North Karoo sub-province (Fig. 1), includes volumetrically minor magma types (Golden Gate, Sani, and Roma units of the Barkly East Formation) [2,10] that have negative DNb values. These are not regarded to record compositional or spatial overlap between North Karoo and South Karoo because of their possible correlation and genetic link to the Ferrar LIP (see discussion in ref. 11). These lavas have been excluded from this study and representative samples listed in Supplementary Table S2 are not plotted in the figures. Some of the dyke samples from the Lesotho area [11] and Theron Mountains [12] have been similarly excluded because of their proclaimed Ferrar-affinity [11,12].

Supplementary figures

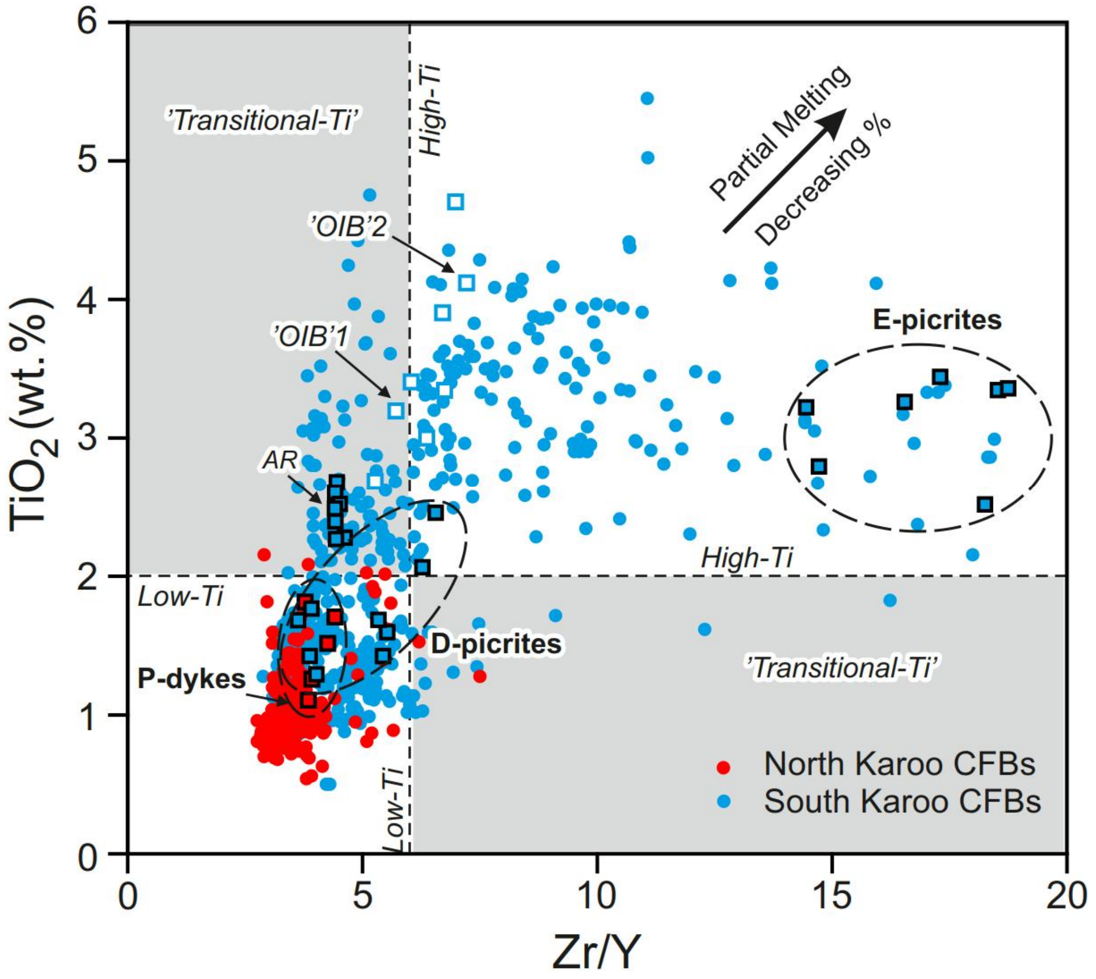

***Supplementary Figure S1*. Conventional classification of Karoo CFBs into low-Ti and high-Ti types.** The Karoo high-Ti CFBs are identified on the basis of TiO_2 >2 wt.% [4] and Zr/Y >6 [13]. Notice that North Karoo CFBs show universal low-Ti affinity, whereas South Karoo CFBs range from low-Ti to high-Ti. CFBs with transitional-Ti compositions (TiO_2 > 2 wt.% and Zr/Y<6 or TiO_2 < 2 wt.% and Zr/Y > 6) are abundant in South Karoo. Plausible parental magma types with MORB-like (D-picrites [7] and Ahlmannryggen (AR) [1] dykes for South Karoo CFBs; P-dykes [6,11] for North Karoo CFBs) and SCLM-like (E-picrites [4,14,15] for South Karoo) characteristics are indicated and compositions of OIB-like South Karoo dykes [1,7] are shown for comparison.

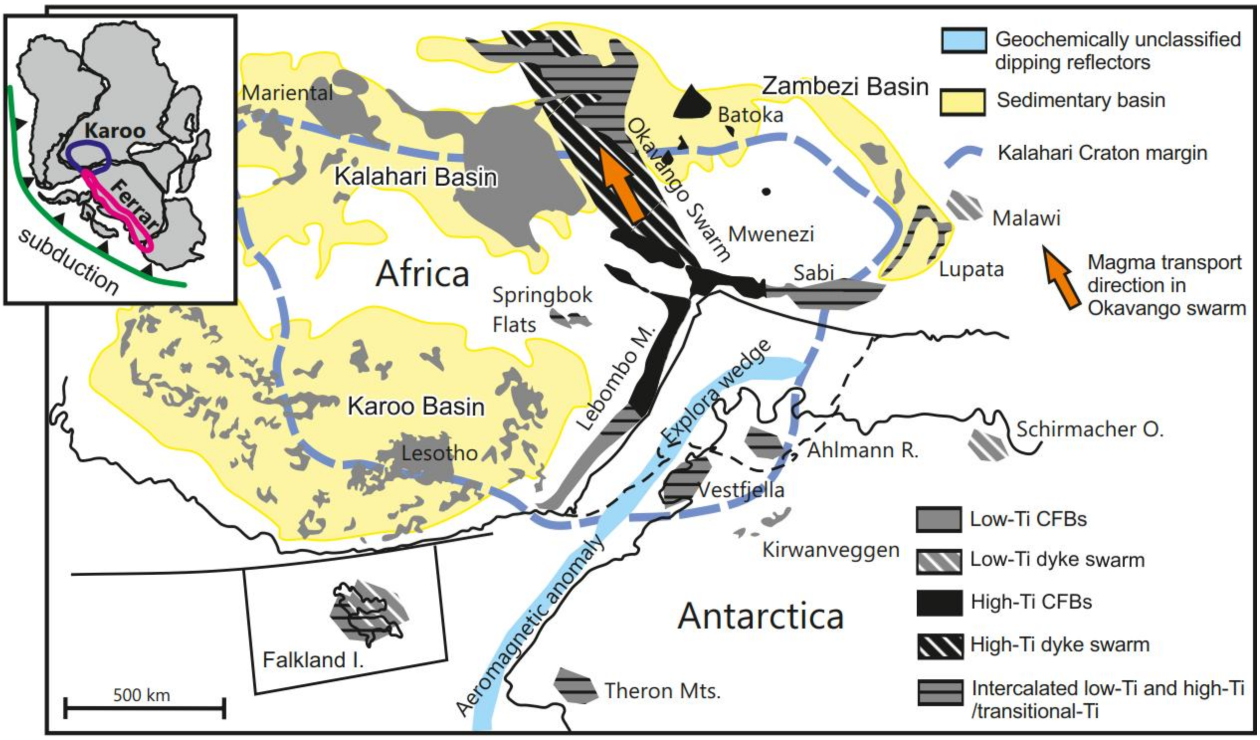

***Supplementary Figure S2*. Distribution of low-Ti and high-Ti (including transitional-Ti) Karoo CFB types in Mid-Jurassic Gondwana reconstruction.** The seaward-dipping lava successions (Lebombo Monocline, Sabi, Vestfjella), radiating dyke swarms (Okavango, Sabi, Lebombo Monocline, Vestfjella, Ahlmannryggen) and submarine seismic reflectors (Explora Wedge) [16] and aeromagnetic anomalies [17] define the Karoo triple rift [5,18]. Distribution of large Permian-Jurassic sedimentary basins in southern Africa and magma transportation direction in the Okavango dyke swarm [5] are indicated. Widespread exposures of CFBs in the Karoo Basin include the Lesotho lava succession and a large sill complex [19]. Notice that high-Ti and transitional -Ti CFBs and dykes are associated with the Karoo triple rift and its vicinity (Batoka, Okavango area, Springbok Flats). Low-Ti CFBs are intercalated with high-Ti and transitional-Ti CFBs across the Karoo rift zone from Lupata to Theron Mountains. Distribution of the Karoo CFBs and the coeval Ferrar CFBs relative to an active subduction zone along southern Gondwana margin is shown in the inset. Occurrences of Karoo-related igneous rocks are after references 4, 16, and 20. The outline of Kalahari Craton [21] is indicated. The map was created using CorelDraw (https://www.coreldraw.com/). The outlines of continents were drawn using vector maps by FreeVectorMaps.com (http://freevectormaps.com) as models and the igneous formations were drawn after Luttinen et al. (ref. 22) and Jourdan et al. (ref. 4) with publisher's permission .

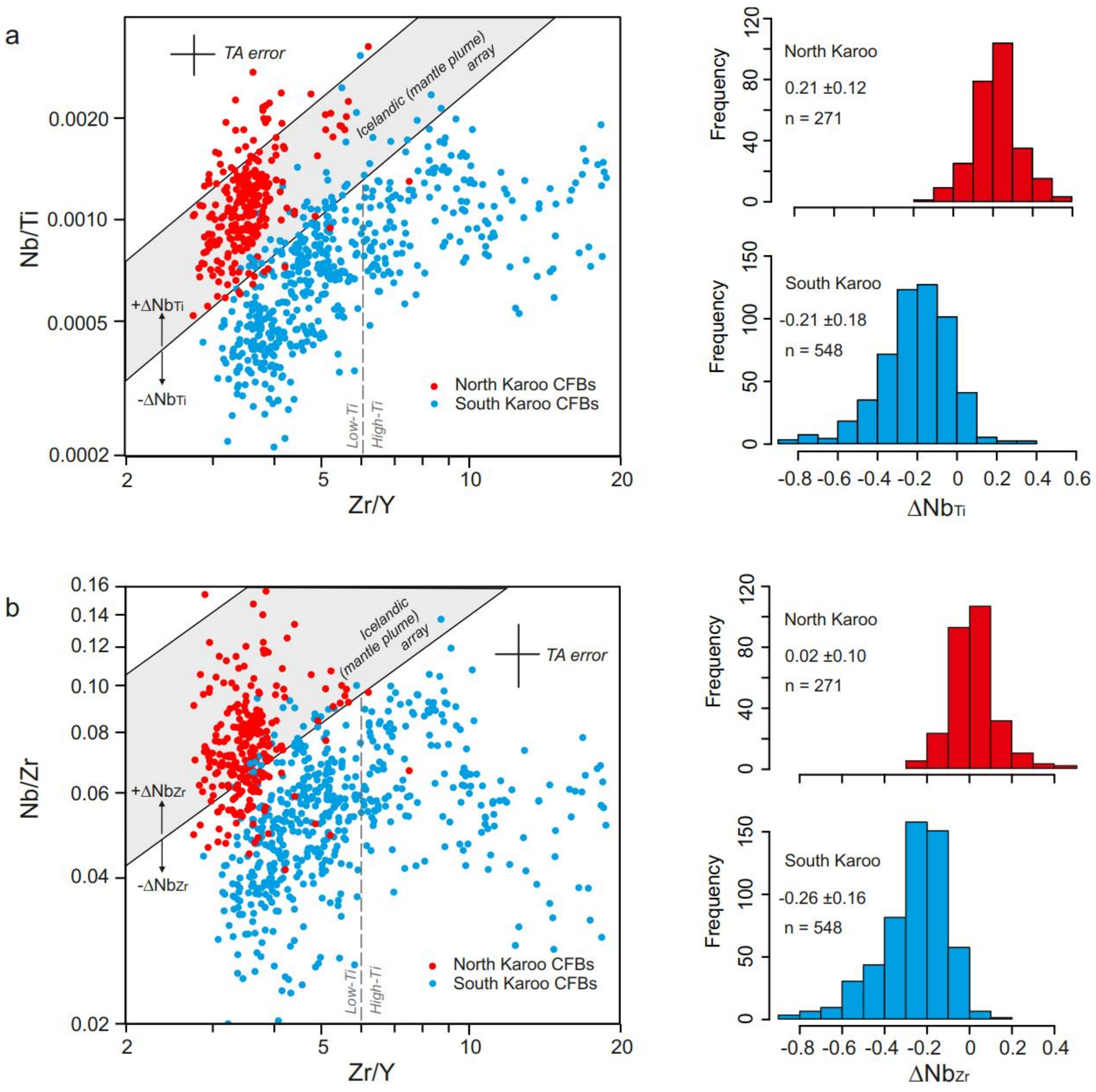

***Supplementary Figure S3*. Relative abundances of Nb, Zr, Ti, and Y in Karoo CFBs and Icelandic plume-derived volcanic rocks.** (a) Variation of Nb/Ti and Z/Y and frequency distribution, average value, and standard deviation of DNb$_{Ti}$. (b) Variations of Nb/Zr and Zr/Y and frequency distribution, average value, and standard deviation of DNb$_{Zr}$. The Icelandic array is from http://georoc.mpch-mainz.gwdg.de/georoc/. For calculation of DNb$_{Ti}$ and DNb$_{Zr}$ and estimated total analytical error (TA error) the reader is referred to Methods.

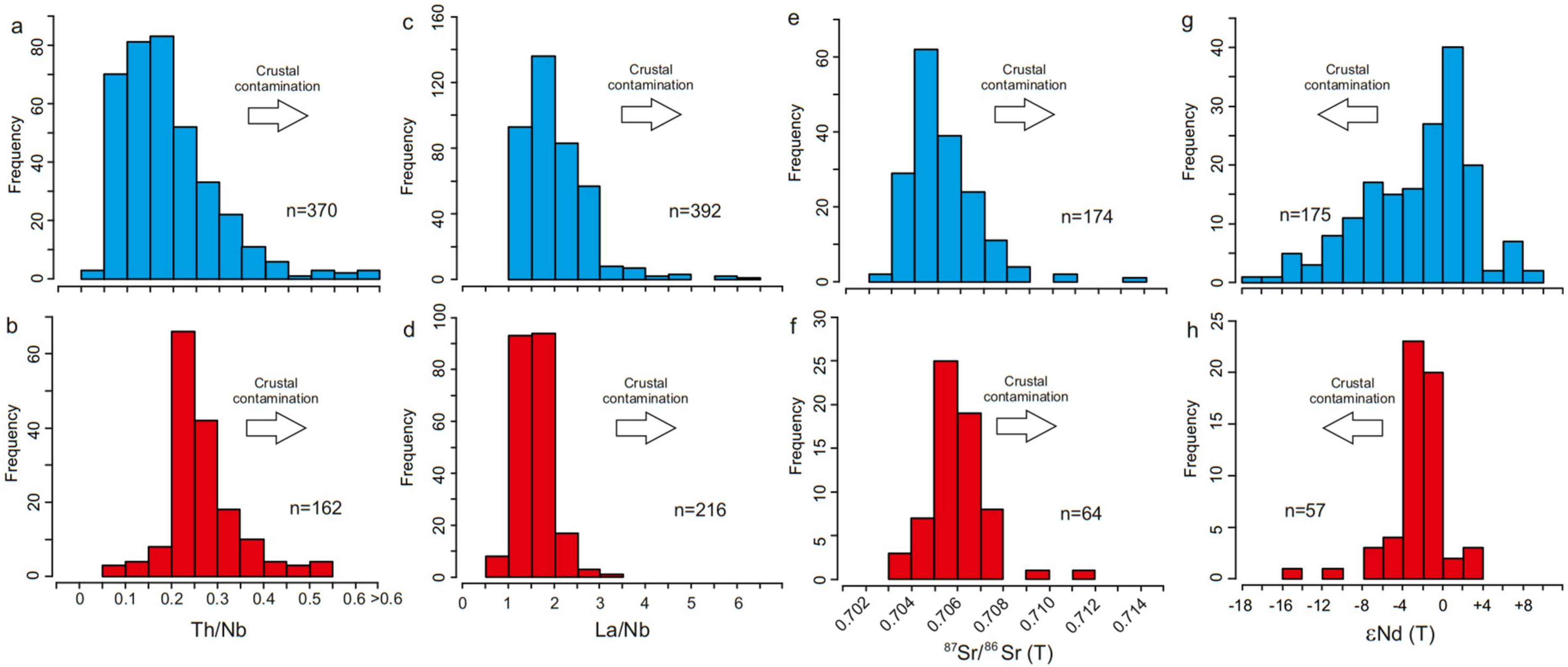

***Supplementary Figure S4*. Frequency distributions for contamination-indexes in Karoo CFBs.** Frequency distribution patterns for (a) Th/Nb in South Karoo, (b) Th/Nb in North Karoo, (c) La/Nb in South Karoo, (d) La/Nb in North Karoo, (e) initial $^{87}Sr/^{86}Sr$ in South Karoo, (f) initial $^{87}Sr/^{86}Sr$ in North Karoo, (g) initial εNd in South Karoo, and (h) initial εNd in North Karoo. The influence of crustal contamination is schematically indicated. Initial isotopic compositions are calculated at 180 Ma.

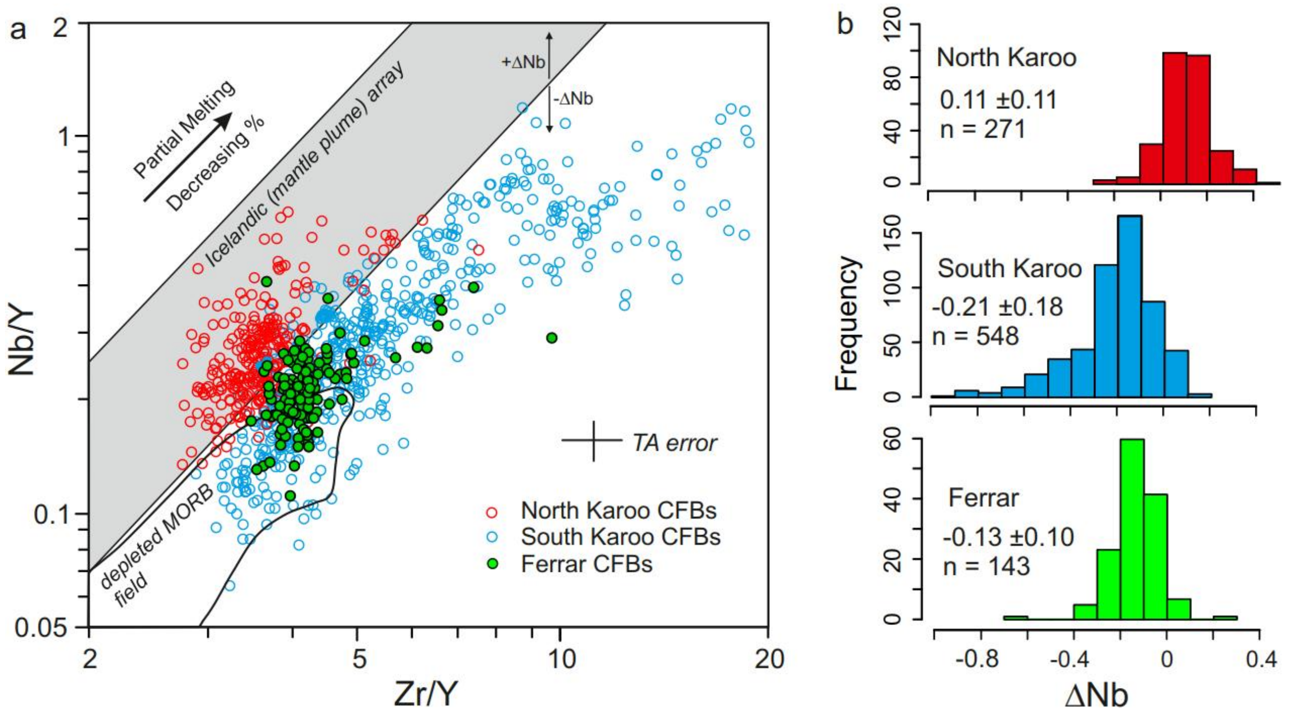

***Supplementary Figure S5*. Relative abundances of Nb, Zr, and Y in Karoo CFBs and Ferrar CFBs.** (a) Variation of Nb/Y and Zr/Y in Karoo CFBs and Ferrar CFBs relative to depleted MORB and Icelandic volcanic rocks. (b) Frequency distributions, average values and standard deviations of DNb in North Karoo, South Karoo, and Ferrar LIP. The Ferrar CFB data are from http://georoc.mpch-mainz.gwdg.de/georoc/. For calculation of DNb and error estimate (TA error) the reader is referred to Methods.

Supplementary references

The geochemical data (n=819) that support the findings of this study include data compiled from references 1–4, 6–12, 14–15, 19–20, 23–37 below (Supplementary Data).

1. Riley, T. R., Leat, P. T., Curtis, M. L., Millar, I. L., Duncan, R. A. & Fazel, A. Early-Middle Jurassic dolerite dykes from Western Dronning Maud Land (Antarctica): Identifying mantle sources in the Karoo Large Igneous Province. J. Petrol. 46, 1489–1524 (2005).
2. Marsh, J. S., Hooper, P. R., Rehácek, J., Duncan, R. A. & Duncan, A. R. in Large Igneous Provinces: Continental, Oceanic, and Planetary Flood Volcanism. (eds Mahoney, J. J. & Coffin, M. F.) 247–272 (American Geophysical Union Monograph 100, 1997).

3. Jones, D. L., Duncan, R. A., Briden, J. C., Randall, D. E. & MacNiocaill, C. Age of the Batoka basalts, northern Zimbabwe, and the duration of Karoo Large Igneous Province magmatism. Geochem. Geophys. Geosyst. 2, 2000GC000110 (2001).
4. Jourdan, F., Bertrand, H., Schaerer, U., Blichert-Toft, J., Féraud, G. & Kampunzu, A. B. Major and trace element and Sr, Nd, Hf, and Pb isotope compositions of the Karoo large igneous province, Botswana-Zimbabwe: lithosphere vs mantle plume contribution. J. Petrol. 48, 1043–1077 (2007).
5. Hastie, W. W., Watkeys, M. K. & Aubourg, C. Magma flow in dyke swarms of the Karoo LIP: Implications for the mantle plume hypothesis. Gond. Res. 25, 736–755 (2014).
6. Hole, M. J., Ellam ,R. M., Macdonald, D. I. M. & Kelley S.P. Gondwana break-up related magmatism in the Falkland Islands. J. Geol. Soc. 173, 108–126 (2015).
7. Heinonen, J. S., Carlson, R. W. & Luttinen, A. V. Isotopic (Sr, Nd, Pb, and Os) composition of highly magnesian dikes of Vestfjella, western Dronning Maud Land, Antarctica: A key to the origins of the Jurassic Karoo large igneous province? Chem. Geol. 277, 227–244 (2010).
8. Luttinen A. V., Heinonen, J. S., Kurhila, M., Jourdan, F., Mänttäri, I., Vuori, S. & Huhma, H. Depleted mantle-sourced CFB magmatism in the Jurassic Africa-Antarctica rift: petrology and 40Ar/39Ar and U/Pb chronology of the Vestfjella dyke swarm, Dronning Maud Land, Antarctica. J. Petrol. 56, 919–952 (2015).
9. Heinonen, J. S., Carlson, R. W., Riley, T. R., Luttinen, A. V. & Horan, M. F. Subduction-modified oceanic crust mixed with a depleted mantle reservoir in the sources of the Karoo continental flood basalt province. Earth Planet. Sci. Lett. 394, 229–241 (2014).
10. Rehácek, J. Chemical and Paleomagnetic Stratigraphy of Basalts in Northern Lesotho, Karoo Province (Unpublished PhD thesis, Washington State University, 1995).

11. Riley, T. R., Curtis, M. L., Leat, P. T., Watkeys, M. K., Duncan, R. A., Millar, I. L. & Owens, W. H. Overlap of Karoo and Ferrar magma types in KwaZulu-Natal, South Africa. J. Petrol. 47, 541–566 (2006).
12. Leat, P. T., Luttinen, A. V., Storey, B. C. & Millar, I. L. in Dyke Swarms: Time Markers of Crustal Evolution (eds Hanski, E. J., Mertanen, S., Rämö, O. T. & Vuollo, J.) 183–199 (Taylor & Francis, London, 2006).
13. Erlank, A.J., Duncan, A. R., Marsh, J. S., Sweeney, R. J., Hawkesworth, C. J., Milner R. McG. & Rogers N. W. A laterally extensive geochemical discontinuity in the subcontinental Gondwana lithosphere. Proceedings of the Geochemical Evolution of the Continental Crust Conference, Pocos de Caldes, Brazil 1–10 (1988).
14. Kamenetsky, V. S. et al. Multiple mantle sources of continental magmatism: Insights from ”high-Ti” picrites of Karoo and other large igneous provinces. Chem. Geol. http://dx.doi.org/10.1016/j.chemgeo.2016.08.034 (2016).
15. Ellam, R. M. & Cox, K. G. An interpretation of Karoo picrite basalts in terms of interaction between asthenospheric magmas and the mantle lithosphere. Earth Planet. Sci. Lett. 105, 330–342 (1991).
16. Jokat, W., Boebel, T., König, M. & Meyer, U. Timing and geometry of early Gondwana breakup, J. Geophys. Res., 108(B9), 2428 (2003).
17. Hunter, R. J., Johnson, A. C. & Aleshkova, N. D. in Weddell Sea tectonics and Gondwana breakup (eds Storey B.C. et al.), 143–154 (Geological Society of London Special Publication 108, 1996).
18. Burke, K. & Dewey, J. F. Plume-generated triple junctions: key indicators in applying plate tectonics to old rocks. J. Geol. 81, 406–433 (1973).

19. Neumann, E., Svensen, H., Galerne, C. Y. & Planke, S. 2011. Multistage Evolution of Dolerites in the Karoo Large Igneous Province, Central South Africa. J. Petrol. 52, 959–984 (2011).
20. Sushchevskaya, N. M., Belyatsky, B. V., Leichenkov, G. L. & Laiba, A. A. Evolution of the Karoo-Maud mantle plume in Antarctica and its influence on the magmatism of the early stages of Indian Ocean opening. Geochem. Int. 47, 1–17 (2009).
21. Jacobs, J., Pisarevsky, S., Thomas, R. J. & Becker, T. The Kalahari craton during the assembly and dispersal of Rodinia. Precamb. Res. 160, 142–158 (2008).
22. Luttinen, A. V., Zhang, X. & Foland, K. A. 159 Ma Kjakebeinet lamproites (Dronning Maud Land, Antarctica) and their implications for Gondwana breakup processes. Geol. Mag. 139, 525–539 (2002).
23. Hawkesworth, C. J., Marsh, J. S., Duncan, A. R., Erlank, A. J. & Norry, M. J. in Petrogenesis of the Volcanic Rocks of the Karoo Province (ed Erlank, A. J.) 341–354 (Geological Society of South Africa Special Publication 13, 1984).
24. Luttinen, A. V. & Furnes, H. Flood basalts of Vestfjella: Jurassic magmatism across an Archaean-Proterozoic lithospheric boundary in Dronning Maud Land, Antarctica. J. Petrol. 41, 1271–1305 (2000).
25. Sweeney, R. J., Duncan, A. R. & Erlank, A. J. Geochemistry and petrogenesis of central Lebombo basalts of the Karoo igneous province. J. Petrol. 35, 95–125 (1994).
26. Luttinen, A. V., Rämö, O. T. & Huhma, H. Neodymium and strontium isotopic and trace element composition of a Mesozoic CFB suite from Dronning Maud Land, Antarctica: implications for lithosphere and asthenosphere contributions to Karoo magmatism. Geochim. Cosmochim. Acta 62, 2701–2714 (1998).

27. Duncan, A. R., Erlank, A. J. & Marsh, J. S. in Petrogenesis of the Volcanic Rocks of the Karoo Province (ed. Erlank, A. J.) 355–388 (Geological Society of South Africa Special Publication 13, 1984)

28. Elburg, M. & Goldberg, A. Age and geochemistry of Karoo dolerite dykes from northeast Botswana. J. S. Afr. Earth Sci. 31, 539–554 (2000).

29. Ellam, R. M. & Cox, K. G. A Proterozoic lithospheric source for Karoo magmatism: evidence from the Nuanetsi picrites. Earth Planet. Sci. Lett. 92, 207–218 (1989).

30. Galerne, C. Y., Neumann E.-R. & Planke, S. Emplacement mechanisms of sill complexes: information from the geochemical architecture of the Golden Valley Sill Complex, South Africa. J. Volcan. Geotherm. Res. 177, 425–440 (2008).

31. Harris, C., Marsh, J. S., Duncan, A. R. & Erlank, A. J. The petrogenesis of the Kirwan Basalts of Dronning Maud Land, Antarctica. J. Petrol. 31, 341–369 (1990).

32. Heinonen, J. S. & Luttinen, A. V. Jurassic dikes of Vestfjella, western Dronning Maud Land, Antarctica: geochemical tracing of ferropicrite sources. Lithos 105, 347–364 (2008).

33. Luttinen, A. V. & Vuori, S. K. in Dyke Swarms - Time Markers of Crustal Evolution (eds Hanski E., Mertanen S. Rämö T. & Vuollo J.) 201–212 (Taylor & Francis Group, London, 2006).

34. Luttinen, A. V., Leat, P. T. & Furnes, H. Björnnutane and Sembberget basalt lavas and the geochemical provinciality of Karoo magmatism in western Dronning Maud Land, Antarctica. J. Volc. Geotherm. Res. 198, 1–18 (2010).

35. MacDonald, R., Crossley, R. & Waterhouse, K. S. Karroo basalts of southern Malawi and their regional petrogenetic significance. Mineral. Mag. 47, 281–289 (1983).

36. Manninen, T. et al. in GTK Consortium Geological Surveys in Mozambique 2002–2007 (eds Pekkala, Y., Lehto, T. & Mäkitie, H.) 211–250 (Geological Survey of Finland Special Paper 48, 2008).

37. Reid, D. L., Rex, D. C. & Brand, G. Karoo basalts in the Ellisras sub-basin, Northern Province. S. Afr. J. Geol. 100, 151–156 (1997).
